# Evaluation of the Bioinductive Effects of a Novel Antibiotic Eluting Cardiac Implantable Electronic Device Envelope

**DOI:** 10.3390/jfb16070234

**Published:** 2025-06-25

**Authors:** Sun Woo Kim, Nathan W. Fedak, Eleanor Love, Alexander Tam, Ali Fatehi Hassanabad, Jeannine Turnbull, Guoqi Teng, Darrell Belke, Justin Deniset, Paul W. M. Fedak

**Affiliations:** Department of Cardiac Sciences, Cumming School of Medicine, University of Calgary, 3330 Hospital Dr. NW, Calgary, AB T2N 2T8, Canada; sun.kim2@ucalgary.ca (S.W.K.); nathan.fedak@gmail.com (N.W.F.); lea.love@mail.utoronto.ca (E.L.); alex.tam1@ucalgary.ca (A.T.); fatehihassanabad@gmail.com (A.F.H.); jdturnbu@ucalgary.ca (J.T.); teng@ucalgary.ca (G.T.); darrellbelke@gmail.com (D.B.); jdeniset@ucalgary.ca (J.D.)

**Keywords:** cardiac implantable device envelope, CIED, antibacterial envelope, biologic envelope, biomaterial-supported drug delivery, rifampin, minocycline

## Abstract

Background: Subcutaneous pocket infection is a common morbidity associated with the integration of cardiac implantable electronic devices (CIEDs). A new antibiotic-eluting CIED bioenvelope has been developed as a prophylactic measure to mitigate infection and skin erosion caused by device migration. This study investigated the envelope’s regulatory properties in scar formation and vascularization. Methods: Fibroblasts were seeded on either plastic (n = 6) or small intestine submucosal extracellular matrix (SIS-ECM) (n = 6) for 24 h. The culture media were analyzed for proangiogenic and proinflammatory proteins with multiplex. Sham (n = 8) or SIS-ECM (n = 8) was randomly implanted into the dorsal subcutaneous pocket of mice. The implants were excised on day 7, cultured for 24 h, and the media analyzed. Rabbit models were implanted with either synthetic polymer HDPE (n = 12) or SIS-ECM (n = 11). The treatments were excised at weeks 2, 10, and 26 and then stained for analysis. Results: SIS-ECM significantly increased the fibroblasts’ paracrine release of proangiogenic and proinflammatory factors like VEGF-A (*p* < 0.05) and IL-6 (*p* < 0.05) compared with plastic. The murine tissue interacting with SIS-ECM released significantly more angiogenic proteins like VEGF-A (*p* < 0.05) than the sham. The histology analysis of rabbit subcutaneous tissue revealed a decreasing level of inflammation and fibrosis over time with SIS-ECM. Conclusions: The CIED bioenvelope elicited proangiogenic paracrine signaling and reduced fibrotic response in fibroblasts and animal models. Clinical translation of the CIED bioenvelope as an adjunct to regular prophylactic practice may be warranted in the future.

## 1. Introduction

Over recent decades, reports of cardiac implantable electronic device (CIED)-related infections have increased dramatically [1]. CIEDs are effective and crucial contemporary therapeutic devices used in managing cardiac arrhythmias [2,3]. However, complications related to CIED device implantations have been increasingly reported [1,2,4,5]. This increasing rate of complications is believed to result from rising life expectancies, the higher rates of CIED implants, and the comorbidities associated with older CIED recipients such as diabetes and renal insufficiency [2,5,6,7,8].

Pocket infections are among the most common CIED-related complications and contribute to high hospitalization costs, reduced quality of life, and increased morbidity and mortality [2,4,9]. Prophylactic measures to reduce post-operative pocket infections have been rigorously studied. Tested interventions include perioperative skin disinfection with iodine or chlorhexidine, irrigation of the subcutaneous pocket with antibiotics before device insertion, and the parenteral administration of antibiotics at different time points [10,11,12,13]. Many of these measures lack evidence supporting their efficacy. CIEDs are intended as lifetime implants, with a frequent need for reoperation that increases the risk of bacterial infection [14]. The lack of long-term prophylactic measures to eradicate bacterial colonies from the local CIED pocket may contribute to increasing hospitalizations. Therefore, developing strategies for the sustained local delivery of antibiotics may improve patient outcomes.

CIED envelopes have been developed to bridge this therapeutic gap. Second generation TYRX (Medtronic Inc., Monmouth Junction, NJ, USA) is a resorbable antibiotic-embedded synthetic mesh prosthesis designed to harbor a CIED generator and resist bacterial infection when implanted in the body [5]. CanGaroo (Elutia Inc., Silver Spring, MD, USA) is a biologic CIED envelope commercially available in North America. Unlike the synthetic TYRX envelope, CanGaroo is a multilayered CIED bioenvelope engineered with decellularized porcine small intestine submucosal extracellular matrix (SIS-ECM) [5]. SIS-ECM contains bioactive factors, such as fibroblast growth factor 2 (FGF-2), which can induce angiogenesis and regulate fibrotic scarring [7]. After the surgical implantation of CIEDs without envelopes, poor tissue regeneration within the pocket can lead to device erosion through the skin and exposure to pathogens [15]. In contrast, excessive fibrotic healing associated with synthetic envelope materials can form a collagenous coating of the device that entraps bacteria that can be later released during reoperation [5]. In previous animal studies, the subcutaneous insertion of the SIS-ECM bioenvelope reduced the infection risk by regulating post-surgical healing around the device. The bioenvelope provides structural support and facilitates vascularization around the device, limiting device migration and directing the local immune response [8,9,16,17].

A novel antibiotic-eluting bioenvelope for CIED has been developed to combine the benefits of local antibiotic delivery with the regenerative properties of SIS-ECM. The EluPro^TM^ Antibiotic Eluting Envelope (Elutia Inc., Silver Spring, MD, USA) is constructed from four layers of porcine SIS-ECM and incorporates a bioabsorbable polymer disc coated with rifampin and minocycline [15]. While the inclusion of the antibiotic polymer in the bioenvelope has proven beneficial, the polymer must not alter the properties of the extracellular material in modulating neoangiogenesis and fibrotic encapsulation. The current study investigated the angiogenic and fibrosis-mediated healing properties of the bioenvelope with antibiotic-eluting discs. First, we assessed the bioinductivity of the extracellular material on fibroblast cells that govern tissue healing. Next, we demonstrated the short- and long-term effects of the antibiotic-eluting bioenvelope on vascularization and fibrotic scarring in mice and rabbit dorsal subcutaneous pockets. We show that the bioinductive properties of SIS-ECM are retained in the new antibiotic-eluting CIED bioenvelope.

## 2. Materials and Methods

### 2.1. CIED Envelope Materials

**Figure 1:**
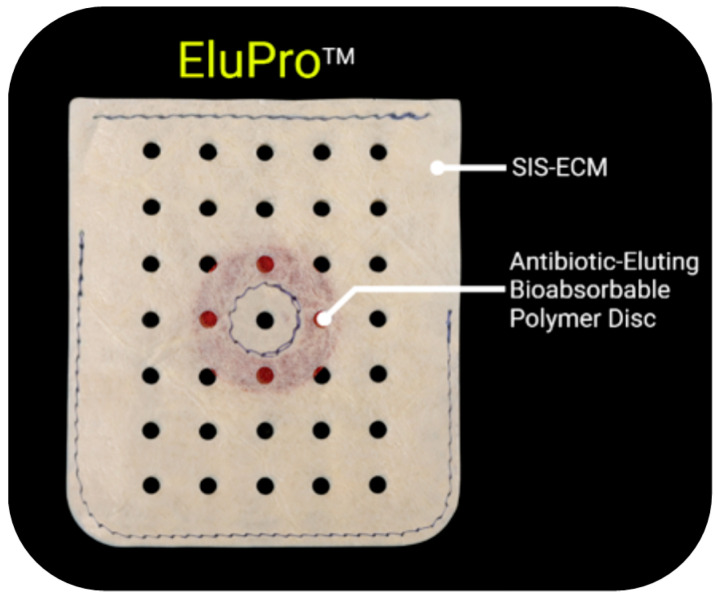
Antibiotic-eluting CIED bioenvelope pouch. The envelope is made of sheets of decellularized, non-crosslinked porcine small intestine submucosa extracellular matrix (SIS-ECM; as labelled) with perforation holes (black dots). The envelope holds an internalized bioabsorbable polymer disc containing the antibiotics rifampin and minocycline. Polymer disc is labelled as Antibiotic-Eluting Bioabsorbable Polymer Disc and highlighted by white line.

The device under study was an antibiotic-eluting bioenvelope called EluPro™ (Elutia, Silver Spring, MD, USA), which is constructed from porcine SIS-ECM with an antibiotic eluting disc adhered to its internal compartment (Figure 1). The SIS-ECM segment of the envelope was isolated and used for our study.

The envelope (6.9 cm × 6.5 cm) was constructed from perforated sheets of decellularized, non-crosslinked, lyophilized ECM derived from porcine intestinal submucosa, combined with discs (diameter: 25 mm) of poly(lactide-co-glycolide) (PLGA), which were engineered to provide extended drug release. The polymer disc was infused with rifampin and minocycline. Rifampin and minocycline used in the formulation were pharmaceutical-grade materials that conformed to United States Pharmacopeia (USP) standards with purities of at least 99%, as determined by high-performance liquid chromatography (HPLC). PLGA has been extensively studied and is widely used as a biodegradable polymer technology for the controlled release of drugs in vivo. In combination with these discs, the bioenvelope was engineered to contain 9.3 mg of minocycline and 10.5 mg of rifampin. Prior studies have demonstrated that antibiotic elution from the device occurs in a biphasic pattern, with an initial bolus followed by sustained release over 2 weeks [15].

### 2.2. Characterization of Eluted Proteins from SIS-ECM

Equal-sized biopsies (~1.4 mm) of the SIS-ECM were excised and soaked in phosphate-buffered saline (PBS) for 3 h ± 20 min. The soaked materials were then placed in a 24-well culture plate with 500 µL Dulbecco’s modified Eagle medium (DMEM) with 0.5% fetal bovine serum (FBS). Plates were incubated at 37 °C with 5% CO_2_ for 24 h (Supplementary Figure S1). The resulting envelope-conditioned media were collected and delivered for blind analysis. Angiogenic protein concentrations were quantified via multiplex (Eve Technologies, Calgary, AB, Canada) and compared with unconditioned cultured media (DMEM with 0.5% FBS).

### 2.3. Murine Fibroblasts Paracrine Response to CIED Envelope Materials

**Figure 2:**
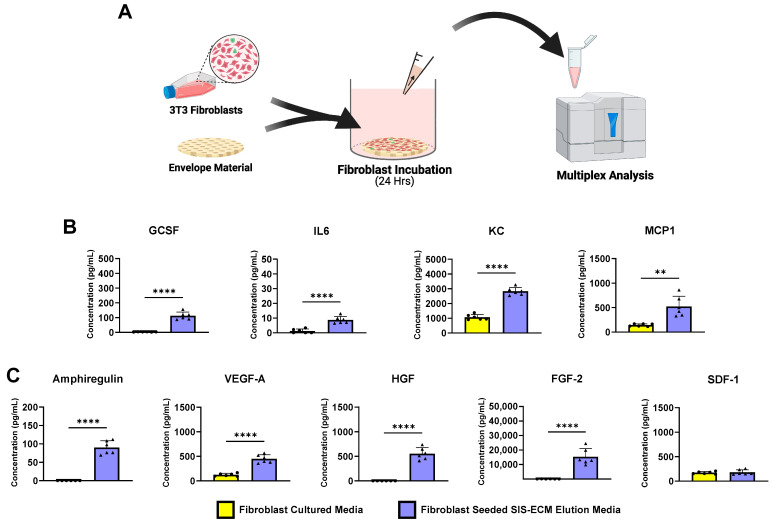
Eluted biofactors from the CIED envelopes and fibroblasts after 24 h. (**A**) Mouse 3T3 fibroblasts were seeded on tissue culture plastic with or without SIS-ECM and incubated for 24 h to generate conditioned media for multiplex analysis. (**B**) Inflammatory and (**C**) angiogenic protein concentrations were measured by the multiplex analysis of the 3T3 cultured media. Plastic served as the control (n = 6, biological replicates) and was compared with SIS-ECM (n = 6, biological replicates). GCSF, granulocyte stimulating factor; IL6, interleukin 6; KC, keratinocyte chemoattractant; MCP1, monocyte chemoattractant protein 1; VEGF-A, vasculogenic endothelial growth factor A; HGF, hepatocyte growth factors; FGF2, fibroblast growth factor 2; SDF-1, stromal cell-derived factor 1. Data are presented as the mean ± SD. ** *p* < 0.01, **** *p* < 0.0001 (two-tail, Student’s T-test).

The envelope materials were prepared in the 24-well culture plates as described. Approximately 400,000 mouse 3T3 fibroblasts were seeded onto either tissue culture-treated polystyrene (plastic; control) or the envelope material and incubated in 500 µL DMEM with 0.5% FBS for 24 h at 37 °C in 5% CO_2_ (Figure 2A). After 24 h, the conditioned media were collected and delivered for blind analysis of the angiogenic protein concentrations by multiplex. The multiplex assay was designed to detect mouse-derived cytokines and growth factors, minimizing the risk for the cross-detection of proteins released from the SIS-ECM, which is derived from pig.

### 2.4. Animals

All animal studies conformed to the Guide for the Care and Use of Laboratory Animals, and the surgical procedures and animal care were conducted at independent research organizations in compliance with Good Laboratory Practice. The Institutional Animal Care and Use Committee approved all animal study protocols before study initiation.

### 2.5. Short-Term Mouse Subcutaneous Healing Study

Male wild type (C57BL/6) mice received implantations of equal-diameter (6 mm) biopsies of SIS-ECM envelopes in surgically created dorsal subcutaneous pockets. As a control, a sham treatment was added, in which blunt dissections were made to generate a subcutaneous pocket, but no material was inserted. Four dorsal pockets were made in 9 mice (36 pockets total) and randomly assigned to the sham or SIS-ECM implantation groups (n = 12/group). On day 7 post-implantation, animals were sacrificed, and the treatments explanted. Adhesion tenacity in each pocket was scored on a 0–5 scale, as shown in Supplementary Figure S2 (0 = No adhesions, no dissection required; 3 = Moderate tenacious adhesions, blunt dissection equal to sharp dissection; 5 = Very tenacious adhesions, sharp dissection only).

**Figure 3:**
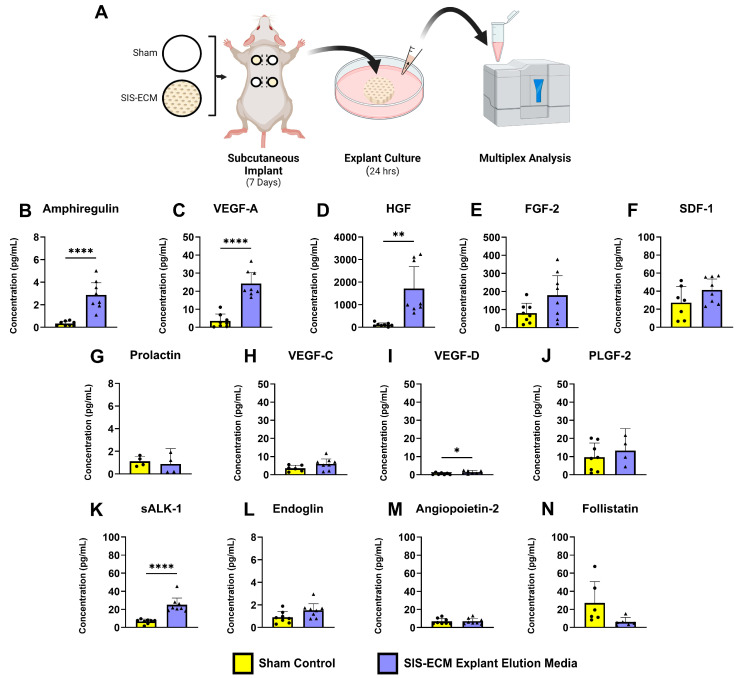
Bioactive factors eluted from the 7-day biopsy explants post 24 h incubation. (**A**) Four dorsal subcutaneous pockets were created in mice and randomly assigned implantation with no biomaterial (sham) or SIS-ECM. Biopsy of the skin and implant was then removed at 7-days and incubated for 24-h to generate conditioned media for multiplex analysis. (**B**–**N**). Sham media served as the control (n = 4–8, biological replicates) and were compared with SIS-ECM (n = 4–8, biological replicates). Data are the presented as mean ± SD. Abbreviations as in Figure 2. * *p* < 0.05, ** *p* < 0.01, **** *p* < 0.0001 (two-tail, Student’s T-test).

The explants and attached tissue (tissue only for the sham group) were weighed and then incubated in 500 uL cell culture media (DMEM with 0.5% FBS) in a 24-well plate for 24 h at 37 °C in 5% CO_2_ (Figure 3A). Angiogenic protein concentrations were quantified using multiplex analysis. Concentrations were normalized to tissue mass (n = 8/group). Normalized protein concentration was calculated by dividing the initial protein concentration by the explant weight minus the dry biomaterial weight: Normalized Protein Concentration = Protein Concentration/(Explant Weight − Dry Biomaterial Weight).

### 2.6. Long-Term Rabbit Subcutaneous Healing Study

**Figure 4:**
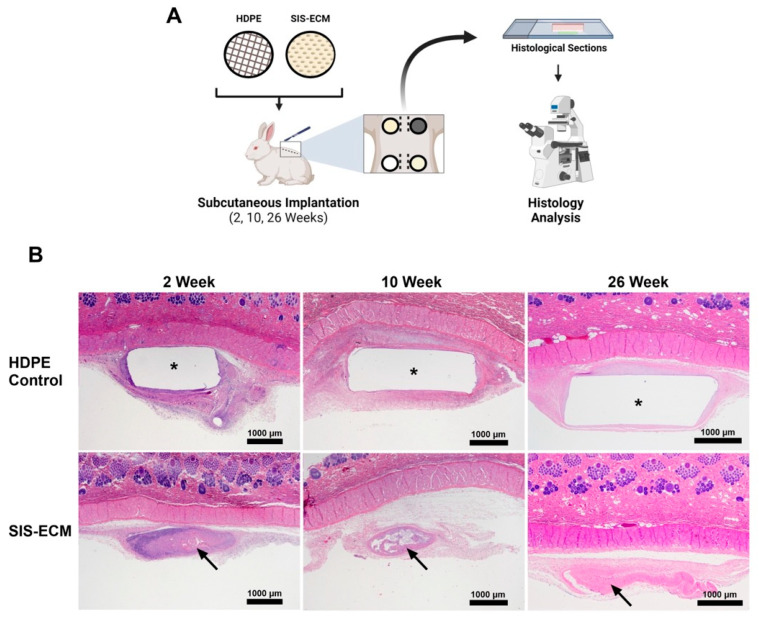
Graphical representation of the long-term healing study with rabbits. (**A**) Rabbits were randomly assigned with HDPE or SIS-ECM subcutaneous implantation and sacrificed at specified time points (2, 10, and 26 weeks) for histological analysis. (**B**) Sample images of hematoxylin and eosin-stained histological sections of tissue explants with either HDPE or SIS-ECM that were used for analysis and scoring. Asterisks (*) represent the HDPE control and the arrows mark SIS-ECM.

New Zealand White rabbits (Charles River Laboratories, Wilmington, MA, USA) received dorsal subcutaneous implantations of equal-diameter (6 mm) biopsies of SIS-ECM envelopes (Figure 4A). As suggested in ISO 10993-6 [18] (Tests for local effects after implantation), high-density polyethylene (HDPE; US Pharmacopeia, negative control plastic) of matching size was used as the negative control. HDPE is a well-established control with good biocompatibility and inert tissue response (ISO 10993, Biological evaluation of medical devices), representing a typical synthetic polymer biomaterial.

Sixteen animals were divided into three groups based on the length of implantation: 2 weeks (n = 5), 10 weeks (n = 5), and 26 weeks (n = 6). Animals were anesthetized with isoflurane gas and received appropriate analgesia. The hair on the back of the rabbit was removed with an electric clipper and the surgical sites aseptically prepared. A skin incision was made parallel to the spine, and a subcutaneous pocket was created by blunt dissection. The SIS-ECM or HDPE control was hydrated with sterile saline for 1–2 min prior to implant. The subcutaneous pockets were closed with 4-O Monocryl suture, and the skin incision was closed as per normal veterinary practice. The animals were given analgesia, kept warm, monitored, and returned to individual housing once they recovered from the anesthesia. Detailed clinical health observations were performed daily on the study animals until termination including the day of termination.

After the designated time points, the animals were euthanized, and necropsy was performed. The envelope and tissue were explanted and fixed in 10% neutral buffered formalin (NBF) for histopathology evaluation.

### 2.7. Histological Analysis of Rabbit Dorsal Subcutaneous Tissue

Tissues were excised and placed in 10% NBF for histology. Formalin-fixed samples were processed by standard histopathology techniques including paraffin embedding, sectioning, and staining with hematoxylin and eosin (Figure 4B). A veterinary pathologist evaluated the samples for the local tissue response to an irritation score from the implanted article as per ISO 10993-6 (2016) (Supplementary Figure S3).

### 2.8. Statistics

GraphPad Prism 10 (GraphPad Software Inc., San Diego, CA, USA) was used for all statistical analysis. Data were analyzed by unpaired Student’s T-test. *p* values < 0.05 were considered significant. All data were expressed as the mean ± SD. Consultation with a professional statistician confirmed that planned analysis of the data using the unpaired T-tests was appropriate.

## 3. Results

### 3.1. SIS-ECM Promotes Murine Fibroblasts to Release Proangiogenic and Proinflammatory Mediators

We aimed to assess the fibroblast phenotype after interaction with SIS-ECM of the bioenvelope. The angiogenic and inflammatory protein expression profiles were evaluated by culturing the cells on SIS-ECM for 24 h and analyzing the media by multiplex analysis (Figure 2A). Media incubated with SIS-ECM without fibroblasts were jointly evaluated to distinguish the proteins eluted from the biomaterial from those released from the biomaterial plus fibroblasts (Supplementary Figure S1).

Compared with fibroblasts cultured on plastic (tissue culture-treated polystyrene), cells that interacted with the SIS-ECM significantly increased their release of the critical proangiogenic proteins amphiregulin, vascular endothelial growth factor A (VEGF-A), hepatocyte growth factor (HGF), and FGF-2 (Figure 2C) (plastic vs. SIS-ECM: VEGFA *p* < 0.0001, HFG *p* < 0.0001, FGF-2 *p* < 0.0001). Moreover, the fibroblasts cultured with SIS-ECM released significantly greater amounts of proinflammatory cytokines involved in wound healing, such as granulocyte-colony stimulating factor (G-CSF), interleukin-6 (IL-6), keratinocyte chemoattractant (KC), and monocyte chemoattractant protein-1 (MCP-1), compared with those cultured on plastic (Figure 2B) (plastic vs. SIS-ECM: G-CSF *p* < 0.0001, IL-6 *p* < 0.0001, KC *p* < 0.0001, MCP-1 *p* = 0.0013). The media from the fibroblast-absent treatment groups had either a lack of or significantly lower concentration of proangiogenic and proinflammatory proteins (Supplementary Figure S1) (plastic vs. SIS-ECM *p* > 0.05).

### 3.2. Mouse Subcutaneous Healing Study

Following the assessment of the bioinductive properties of SIS-ECM on the murine fibroblasts, we next evaluated whether the angiogenic properties could be replicated in mice after subcutaneous implantation. Seven days after implantation in the dorsal subcutaneous pocket, the biomaterial and any adhered tissues were explanted and incubated in low-serum media for 24 h (Figure 3A). The media were then analyzed via multiplex analysis to measure the release of angiogenic growth factors from the surgical site. The media cultured with skin explant from the sham surgical sites were used as a control.

Compared with the media cultured with sham tissue, the media cultured with the murine tissue with SIS-ECM contained significantly higher levels of the angiogenic proteins amphiregulin, sALK-1, VEGFA, and HGF (Figure 3B–N) (sham vs. SIS-ECM: amphiregulin *p* < 0.0001, sALK-1 *p* < 0.0001, VEGFA *p* < 0.0001, HGF *p* = 0.0016).

### 3.3. Long-Term Healing Study in Rabbits

**Figure 5:**
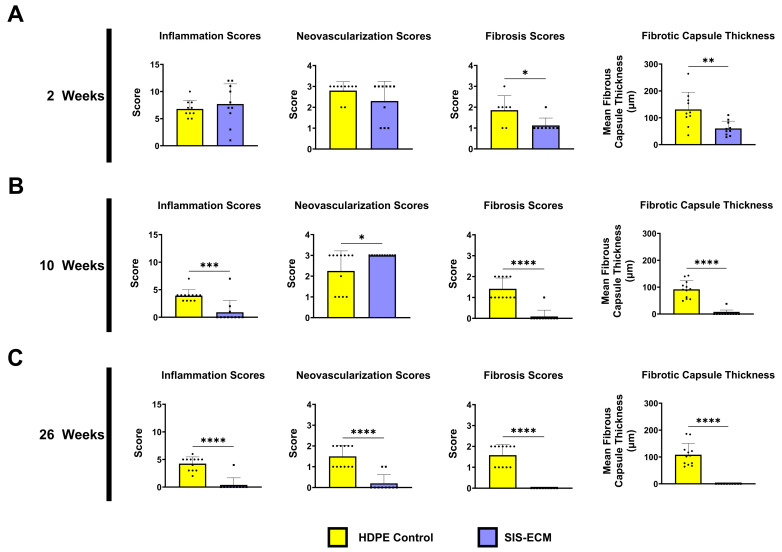
Microscopic scores of inflammation and tissue response of the histological sections of rabbit dorsal subcutaneous tissue at 2, 10, and 26 weeks. (**A**) Rabbits were randomly subjected to either high-density polyethylene (HDPE) or SIS-ECM in one of the four dorsal subcutaneous pockets. Inflammation, neovascularization, fibrosis score, and fibrotic capsule thickness around the HDPE (n = 7–12 biological replicates) and SIS-ECM (n = 8–11 biological replicates) were measured at week 2 (**A**) and subsequent measures for the week 10 (**B**) and week 26 explants (**C**). Data are presented as the mean ± SD. * *p* < 0.05, ** *p* < 0.01, *** *p* < 0.001 **** *p* < 0.0001 (two-tail, Student’s T-test).

Wound healing responses to the implanted biomaterial were studied over the long-term (26 weeks) in the rabbit subcutaneous model, which compared SIS-ECM to HDPE (Figure 4). Hematoxylin and eosin-stained histological sections of the explanted tissues showed evidence of constructive remodeling in the SIS-ECM explants compared with the complete absence of tissue ingrowth in the HDPE group (Figure 4B). A clear fibrosis capsule was observed around the HDPE throughout the 26-week period, but not around the SIS-ECM. Tissue responses to SIS-ECM showed an adaptive wound healing process, with an initial infiltration of immune cells, such as lymphocytes and macrophages, observed at early time points (immune cell histological score 8.5 at 2 weeks) (Figure 5A) (HDPE vs. SIS-ECM: inflammation *p* = 0.488). The initial inflammatory phase resolved quickly around SIS-ECM, and significantly fewer immune cells were observed at 10 weeks (immune cell score 1.0 at 10 weeks) (Figure 5B) (HDPE vs. SIS-ECM: inflammation *p* = 0.0003). By 26 weeks, immune cells were almost absent around SIS-ECM (score 0.4 at 26 weeks), indicating a mature wound healing process (Figure 5C) (HDPE vs. SIS-ECM: inflammation *p* < 0.0001). However, inflammation remained unresolved around the synthetic polymer material throughout the 26 weeks. The presence of immune cells, including polymorphonuclear cells, macrophages, and multinucleated giant cells, was evident in both week 10 and week 26. The immune cell histological scores increased slightly from 3.9 at 10 weeks to 4.3 at 26 weeks, suggesting a sustained foreign body response.

Angiogenesis around and into SIS-ECM biomaterials followed the expected adaptive wound-healing cascade. A relatively high level of neovascularization was observed at early time points (neovascular histological score of 2.3 at 2 weeks and 3.0 at 10 weeks). Neovascular formation around SIS-ECM was absent by 26 weeks (score of 0.2), indicating the maturation of blood vessels around the implant. In contrast, neovascularization persisted around the synthetic polymer material for up to 26 weeks. The neovascular histological score around HDPE declined slowly over time but remained at a relatively high level of 1.5 at 26 weeks. This finding is consistent with unresolved local inflammation and prolonged foreign body response observed around the synthetic material (Figure 5).

To compare the extent of fibrosis tissue formation surrounding the two different materials, histological scores were used to measure the extent of fibrosis. At 2 weeks, the fibrosis histological score was significantly lower for SIS-ECM (1.1) compared with HDPE (1.9) (HDPE vs. SIS-ECM: fibrosis score *p* = 0.0204). Over time, the fibrotic tissue around SIS-ECM almost completely resolved, with scores of 0.1 at 10 weeks and 0 at 26 weeks (HDPE vs. SIS-ECM: fibrosis score *p* < 0.0001). In contrast, fibrosis persisted around the synthetic polymer material (HDPE), with scores of 1.4 at 10 weeks and 1.6 at 26 weeks.

The capsule thickness around the implant further demonstrated the significant difference in fibrosis between SIS-ECM and the synthetic material. At 2 weeks, the average capsule thickness around SIS-ECM was only half of that around the synthetic mesh (60 µm vs. 131 µm) (HDPE vs. SIS-ECM: fibrotic capsule thickness *p* = 0.0049). At 10 weeks, the average thickness of the capsule around SIS-ECM decreased to 3.5 µm (HDPE vs. SIS-ECM: fibrotic capsule thickness *p* < 0.0001). By 26 weeks, no fibrotic capsule was present around the SIS-ECM material (HDPE vs. SIS-ECM: fibrotic capsule thickness *p* < 0.0001). In contrast, the fibrotic capsule persisted around the synthetic material, with no sign of decreasing (92 µm at 10 weeks and 108 µm at 26 weeks). These findings underscore the regenerative properties of SIS-ECM, as evidenced by significantly reduced fibrosis and capsule formation compared with the synthetic material.

## 4. Discussion

The incidence of CIED-related infections has risen with the increasing number of CIED implants. Pocket infections remain the most prevalent complication associated with CIEDs, and recent studies have focused on identifying the source of bacteria causing the pocket infections to support targeted prophylactic measures. In one study, bacteriologic specimens were collected from the device pocket before and after generator insertion and compared with specimens from the neighboring pre-axillary skin flora [19]. The group verified that 60% of recipients who developed a pocket infection had positive cultures, including staphylococci, enterococci, and streptococci species, highlighting the role of bacterial migration from the skin as a potential source of infection [5]. Other studies have reported that poor wound healing can lead to the opening of the surgical area through device erosion, allowing for bacterial infiltration [15,20]. There are also risks from reoperation and hematogenous seeding from remote sources of infection [8,20]. Several groups have verified that Gram-positive species like staphylococci largely accounted for these infections, with complications often involving antimicrobial-resistant Gram-negative species like Hemophilus influenzae. If left untreated, bacteria from pocket infections can propagate to the intravenous leads and develop into bacteremia or endocarditis [10,21].

Prophylactic measures to target pocket infections have been studied, but few have sufficient evidence to support routine clinical use. In one study, preoperative intravenous administration of cefazolin reduced the rate of infections within 6 months of follow-up. Since this study, the administration of cefazolin has become a common prophylactic practice before CIED insertion [10,22]. However, CIEDs are intended as lifetime implants that may require regular reoperations, each of which increases the risk for bacterial infiltration. Antibiotic-eluting CIED envelopes have proven effective as a long-term intervention against pocket infections. For example, TYRX is a synthetic, absorbable mesh envelope that releases rifampin and minocycline for at least 7 days to inhibit the proliferation and growth of a wide spectrum of bacteria including the Gram-positive species commonly found in pocket infections. The efficacy of this antibiotic-eluting envelope in reducing pocket infection has been demonstrated by several studies, including the Worldwide Randomized Antibiotic Envelope Infection Prevention Trial (WRAP IT), which assessed the efficacy of the bioabsorbable envelope in reducing CIED infection 12 months after the implant procedure [23]. A total of 6983 subjects from 25 countries undergoing pocket revisions, generator replacements or upgrades, and initial cardiac defibrillator implantation were randomly assigned to the envelope or control (no envelope). The study found that the antibacterial envelope reduced CIED-related infections by 40% when used adjunctively with standard-of-care infection-prevention strategies [23].

A key limitation of synthetic surgical materials, however, is their propensity to elicit a robust foreign body response, leading to fibrous encapsulation of the device [5,9]. In contrast, non-crosslinked biologic materials, such as SIS-ECM, interact with host cells to moderate the inflammatory response and promote neoangiogenesis and the ingrowth of site-appropriate native tissues into the matrix [5,24,25]. The resulting vascularized pocket may improve host immune access to the tissues surrounding the device as well as facilitating future reoperations [26,27,28]. A novel antibiotic-eluting bioenvelope for CIED has been developed to demonstrate the combined effects of local antibiotic delivery with the regulatory function of SIS-ECM (Figure 1). In a recent study, the antibacterial efficacy of this envelope was evaluated in New Zealand White rabbits through the implantation of either the antibiotic-eluting bioenvelope or control material (envelope without antibiotics) in surgically generated dorsal subcutaneous pockets [15]. The pockets were then injected with one of four strains of bacteria commonly found in pocket infections. During the 7-day daily check-up and post-7-day autopsy, animals that received the antibiotic-eluting bioenvelope showed no signs of infection, regardless of the species of bacteria injected.

The current study evaluated the effects of this antibiotic-eluting SIS-ECM bioenvelope on the mechanisms of tissue regeneration and healing. Extensive literature has documented the constructive remodeling of intact ECM when implanted in vivo, including neoangiogenesis, an abbreviated inflammatory response, incorporation into native tissues, and the absence of fibrotic encapsulation [8,17,24]. The key question addressed by this study was whether adding a drug-eluting PLGA disc to the SIS-ECM would alter the tissue healing response associated with intact ECM. Our findings suggest that the PLGA disc does not interfere with the normal wound healing fostered by SIS-ECM. Proangiogenic proteins, growth factors, and other bioactive factors associated with constructive tissue remodeling were released when the bioenvelope was cultured with murine fibroblasts and from bioenvelopes excised after in vivo implantation (Figure 2 and Figure 3). Explanted bioenvelopes also showed minimal fibrosis or adhesion formation (Figure 5) (Supplementary Figure S2).

Previous studies have described a reciprocal relationship between implanted ECM and host tissues. In vitro, growth factors and other bioactive components retained within the SIS-ECM are released from the device by simple diffusion. In vivo, however, the release of bioactive factors is promoted by degradation of the ECM by host enzymes [25]. Following implantation, or when placed in a culture with appropriate cell types, intact ECM stimulates the release of enzymes that cleave ECM components, leading to the release of embedded bioactive factors that, in turn, influence cellular behavior and phenotype [24]. Host fibroblasts surrounding the SIS-ECM secrete matrix metalloproteinases (MMPs), particularly MMP-2 and MMP-9, which degrade collagen and other matrix proteins, facilitating the release of the bioactive factors. In prior studies, the mediators and growth factors released from implanted ECM induced a proangiogenic and proinflammatory fibroblast phenotype that stabilized fibroblast-mediated remodeling of endogenous ECM in damaged tissue [26]. This reciprocity between ECM and fibroblasts was further demonstrated in the current study. When the bioenvelope was placed in culture media without fibroblasts or other tissues, it did not release significant levels of bioactive factors (Supplementary Figure S1), indicating that these effects require interaction with appropriate cell types. In contrast, the coculturing of murine fibroblasts with the ECM bioenvelope promoted a shift in cell phenotype, with an elevated release of angiogenic proteins (VEGF-A, HGF, and FGF-2) and proinflammatory cytokines (G-CSF, IL-6, KC, and MCP1) (Figure 2).

Analysis of the explanted bioenvelopes from the rabbit subcutaneous implant model illustrated key steps in the normal tissue healing process. At 2 weeks post-implantation, the inflammation and neovascularization scores were elevated, reflecting an acute wound-healing response (Figure 5A). By week 10, acute inflammation had waned, but neovascularization scores remained elevated as the proangiogenic factors released from the SIS-ECM continued to promote vascularization (Figure 5B). By week 26, the inflammation, neovascularization, and fibrosis scores were near zero in the bioenvelope group, consistent with a completed healing process and incorporation into native tissues (Figure 5C). In contrast, the explanted synthetic material (HDPE) demonstrated persistently elevated inflammation and fibrosis scores and developed a thick fibrotic capsule (Figure 5).

Evaluation of the adhesion tenacity of the SIS-ECM envelope 7 days after in vivo implantation demonstrated a moderate degree of adhesion tenacity when compared with the sham connective tissue (Supplementary Figure S2). Along with the reduced thickness of the fibrotic capsule in rabbits over time (Figure 5), these findings align with the results of previous studies of ECM-based biomaterials and indicate that the antibiotic-eluting bioenvelope induces healthy tissue healing, regardless of the presence of the antibiotic-coated PLGA disc [24,25,27,28,29]. Importantly, the ability of the device to integrate into native tissues supports the long-term performance of the bioenvelope in limiting the formation of adhesions and the persistence of bacteria trapped within the pocket [25]. Therefore, the antibiotic-eluting bioenvelope may promote the clearance of bacteria from the pocket as well as providing the local delivery of clinically meaningful levels of the broad-spectrum antibiotics rifampin and minocycline. Furthermore, as demonstrated by previous studies, the antibiotics were released from the device in a biphasic pattern, providing an initial bolus of drug elution, followed by more gradual release over 2 weeks [15]. The long-term in vivo experiments conducted as part of the current study, therefore, encompass this timeframe of antibiotic release from the envelope. The qualitative and quantitative findings describing constructive remodeling with the antibiotic-eluting SIS-ECM during this timeframe indicate that the release of antibiotics has, at most, a minimal impact on the remodeling of SIS-ECM.

Evidence from clinical studies further illustrates the regenerative qualities and clinical benefits of the SIS-ECM bioenvelope. In one study, patients who received the bioenvelopes (without antibiotic-eluting PLGA discs) during their initial CIED surgery had easier reoperations on average, with fewer lead adhesions and less capsulectomy during subsequent procedures compared with those who did not receive an envelope or who received the non-biologic envelope [28]. In fact, histologic analysis from a recent case study demonstrated that the use of the bioenvelope in a patient with an established dense fibrotic capsule led to the rejuvenation of the device pocket through the regeneration of healthy, vascularized tissue around the device [29].

### 4.1. Limitations

This study had several important limitations. First, the study lacked certain controls such as the evaluation of PLGA discs without antibiotics, SIS-ECM bioenvelopes without PLGA discs, and synthetic antibiotic-eluting CIED envelopes. These materials were excluded from the experiments because they are proprietary and therefore have restricted access. Ideally, future studies would include these control materials to strengthen the analysis and distinguish the bioinductive effects of the antibiotic-eluting bioenvelope from its individual components. Head-to-head preclinical and clinical studies are also warranted to evaluate the comparative performance of the antibiotic-eluting bioenvelope and the synthetic envelope. Second, the study did not evaluate the characteristics of the PLGA disc, such as wettability, which could influence the healing response. Third, the in vivo studies included time points at 2, 10, and 26 weeks; additional intermediate time points (e.g., 18 weeks) could offer further insights into the dynamics of bioinduction. The included time points were selected based on logistical considerations and the recommendations of standards such as ISO 10993-6 [18].

### 4.2. Conclusions

This study demonstrates that the SIS-ECM bioenvelope combined with an antibiotic-eluting PLGA disc promoted the release of critical proangiogenic and pro-remodeling factors that support adaptive wound healing and neovascularization, limit chronic inflammation, and minimize fibrosis of the implant. Together with recent data demonstrating the antimicrobial efficacy of this device [15], our results support the clinical utility of the antibiotic-eluting bioenvelope for securing devices, such as CIED, while integrating into host tissues, promoting long-term performance, and minimizing risk for infection and other adverse events.

## Data Availability

The raw data supporting this article will be made available by the corresponding author on request.

## Supplementary Materials

The following supporting information can be downloaded at: https://www.mdpi.com/article/10.3390/jfb16070234/s1, Figure S1. Schematic representation of generating conditioned media for analysis (**A**). Multiplex analysis of inflammatory (**B**) and angiogenic (**C**) proteins of the biomaterial conditioned media, plastic media control (n = 6, biological replicates), and SIS-ECM (n = 6, biological replicates). Abbreviations as in Figure 2. Data are presented as the mean ± SD. * *p* < 0.05, ** *p* < 0.01 (two-tail, Student’s T-test). Figure S2. Schematic representation of the adhesion assessment of the implants at 7 day in mice (**A**). Sample images of the implants (**B**). Assessment criteria for adhesion scoring (**C**). Adhesion tenacity scores of the sham (n = 12, biological replicates) and SIS-ECM (n = 12, biological replicates) (**D**). Data are presented as the mean ± SD. **** *p* < 0.0001 (two-tail, Student’s T-test). Figure S3. Assessment criteria for scoring inflammation, neovascularization, and fibrosis in the long-term subcutaneous healing study of rabbits at weeks 2, 10, and 26.
