# Supplementary material for: Evaluation of the Bioinductive Effects of a Novel Antibiotic Eluting Cardiac Implantable Electronic Device Envelope
Source: J Funct Biomater. 2025 Jun 25;16(7):234. doi: 10.3390/jfb16070234 (PMC12295061; doi:10.3390/jfb16070234)
Supplement: Supplementary file 1 [file jfb-16-00234-s001.zip › jfb-3658429-supplementary.pdf]

Supplementary Figures

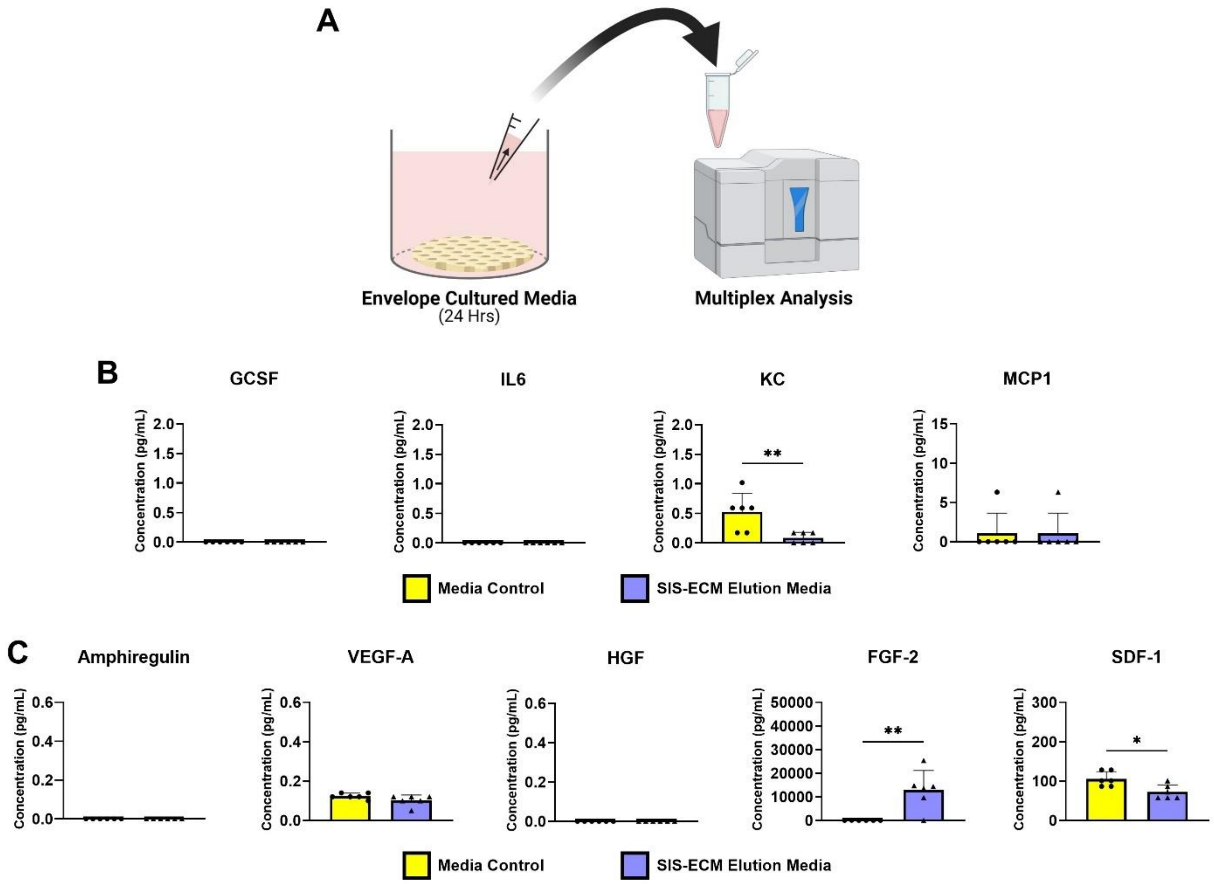

Supplementary Figure S1. Schematic representation of generating conditioned media for analysis (A). Multiplex analysis of inflammatory (B) and angiogenic (C) proteins of the biomaterial conditioned media Plastic Media Control (n = 6, Biological Replicates) and SIS-ECM (n = 6, Biological Replicates). Abbreviations as in Figure 2. Data are presented as mean ± SD. *$p < 0.05$, **$p < 0.01$ (Two Tail, Student's T-Test)

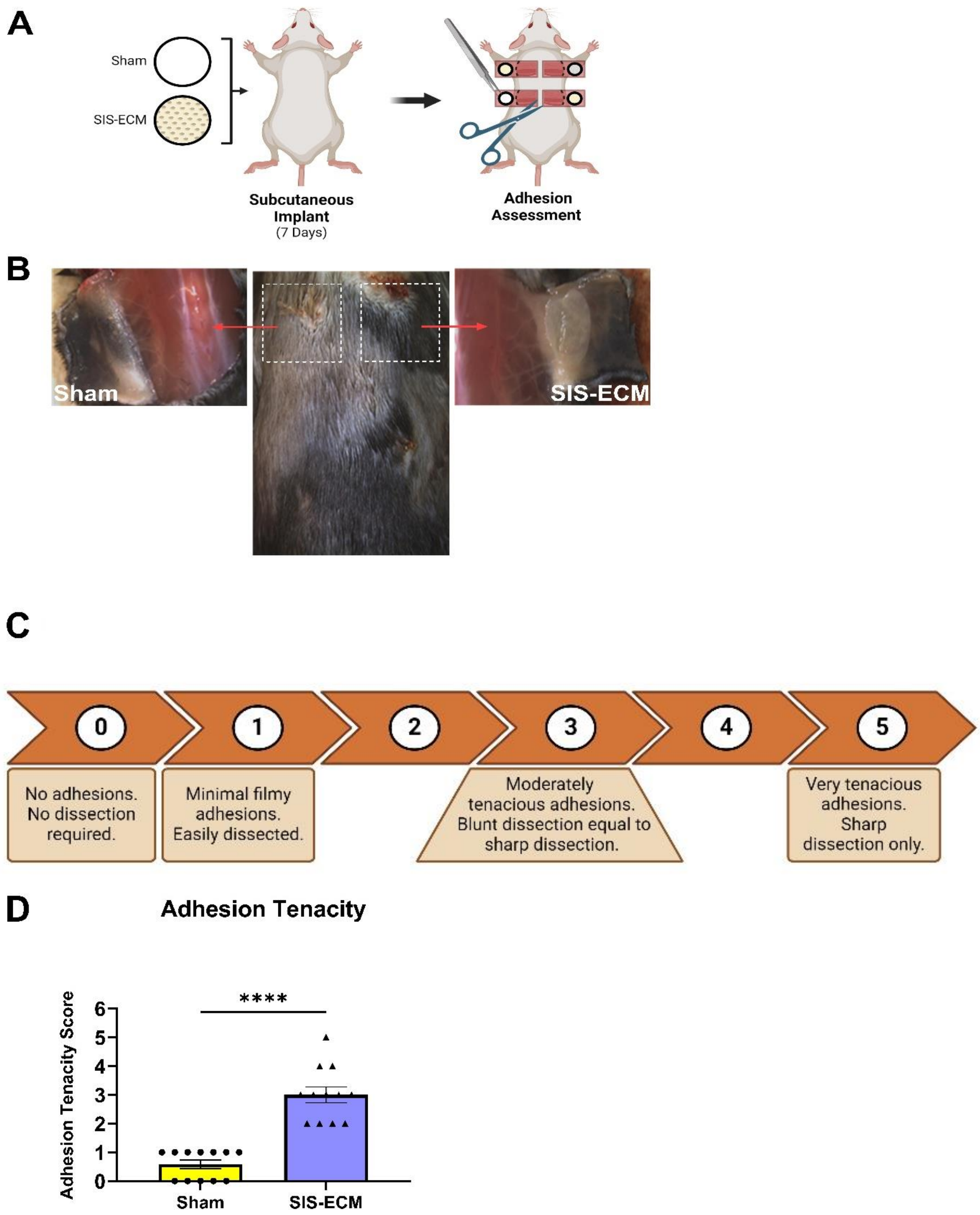

Supplementary Figure S2. Schematic representation of the adhesion assessment of the implants at 7 day in mice (A). Sample images of the implants (B). Assessment criteria for adhesion scoring (C). Adhesion tenacity scores of Sham (n = 12, Biological Replicates) and SIS-ECM (n = 12, Biological Replicates) (D). Data are presented as mean ± SD. **** $p < 0.0001$ (Two Tail, Student's T-Test).

KEY FOR TABLES 1 - 4:					
Group Average: The implant scores [I + TR] for each implant site scored are totaled. The Group Average = the Sum of the Total scores for that Group, divided by the number of implant sites, rounded to the nearest 10th decimal point.					
NA = not applicable. ID = Identification.<br>Evaluation includes original and all re-cut, re-trimmed, and/or re-embedded slides, as applicable, unless otherwise indicated.					
**Severity Scores (per implant site):**					
	**Score**				
**Inflammation**	**0**	**1**	**2**	**3**	**4**
Polymorphonuclear cells (PMNs)<br>Lymphocytes<br>Plasma Cells<br>Eosinophils<br>Macrophages	Absent: 0	Minimal: Rare, 1-5/HPF	Mild: 6-10/HPF	Moderate: Heavy Infiltrate	Marked/Severe: Packed
Multinucleated Giant Cells	0	Rare, 1-2/HPF	3-5/HPF	Heavy Infiltrate	Sheets
Necrosis [a]	0	Minimal	Mild	Moderate	Marked/Severe
HPF = high powered field (40X), averaged over the entire implant site					
	**Score**				
**Tissue Response**	**0**	**1**	**2**	**3**	**4**
Neovascularization	Absent: None	Minimal: Minimal capillary proliferation, (focal, 1-3 capillary buds), or small blood vessels (venules, and / or arterioles)	Mild: Groups of 4-7 capillaries with supporting fibroblastic structures	Moderate: Broad band of capillaries with supporting structures	Marked/Severe: Extensive band of capillaries with supporting fibroblastic structures
Fibrosis / Fibrous Capsule [b]	Absent: Fibrous capsule is not formed (i.e.: cellular capsule composed predominantly of inflammatory cells with little fibroplasia), or is too thin to measure	Minimal: Narrow band (≤100 μm Mean Capsule Thickness)	Mild: Moderately thick band (>100, up to 200 μm Mean Capsule Thickness)	Moderate: Thick band (>200, up to 300 μm Mean Capsule Thickness)	Marked/Severe: Extensive band (>300 μm Mean Capsule Thickness)
	**Score [c]**				
	**0**	**1**	**2**	**3**	**4**
Granuloma Formation<br>Traumatic Myofiber Changes<br>Fatty Infiltration<br>Bone Formation [d]<br>Tissue Ingrowth into the Implanted Material<br>Soft Tissue Mineralization<br>Hemorrhage, subacute / chronic [e]<br>Foreign Material (other than implant material)	Absent: None	Minimal: > 0 up to 25% of the implant field	Mild: > 25 up to 50% of the implant field	Moderate: > 50 up to 75% of the implant field	Marked/Severe: > 75 up to 100% of the implant field
[a] Necrosis consists of nuclear cellular debris from degenerating inflammatory cells.					
[b] The fibrous capsule score is derived from the average of the mean capsule thickness (in microns) for each of the implant sites.					
[c] Percentages of tissue component(s) are estimates.					
[d] Criteria for bone formation includes the presence of one or more of the following features: a) cartilage, b) chondrocytes / chondroblasts, c) new bone / osteoid, d) osteoblasts / osteocytes, and/or, e) bone marrow.					
[e] Acute hemorrhage is not graded. Acute hemorrhage is generally due to rupture of new blood vessels within the tissue reaction from tissue handling.					
Fibrous Capsule Measurements:<br>M1 = measurement 1; M2 = measurement 2; M3 = measurement 3. (μm = microns). Measurements of the thickness (in μm) of the fibrous capsule are estimated. This measurement is an estimate of the width of the capsule around the implant, but excludes the actual implant material. The capsule measurements are taken at three different representative places, and the mean is the average of these three measurements. Measurements are recorded as "0" if the capsule is not formed, or is too thin to measure.					

Supplementary Figure S3. Assessment criteria for scoring inflammation, neovascularization, and fibrosis in the long-term subcutaneous healing study of rabbits at weeks 2, 10, and 26.
